# 17.3 EFFECT OF CANNABIDIOL ON SYMPTOMS, DISTRESS AND NEUROPHYSIOLOGICAL ABNORMALITIES IN CLINICAL HIGH-RISK FOR PSYCHOSIS PATIENTS: A PLACEBO-CONTROLLED STUDY

**DOI:** 10.1093/schbul/sby014.067

**Published:** 2018-04-01

**Authors:** Sagnik Bhattacharyya, Robin Wilson, Paul Allen, Matthijs Bossong, Elizabeth Appiah-Kusi, Philip McGuire

**Affiliations:** 1 Institute of Psychiatry, Psychology & Neuroscience, King’s College London; 2 Brain Center Rudolf Magnus, University Medical Center Utrecht

## Abstract

**Background:**

There has been growing interest in the therapeutic potential of Cannabidiol (CBD) stemming from independent evidence that CBD has antipsychotic and anxiolytic properties in patients with mental health disorders. CBD has been found to be non-inferior to antipsychotic medication in a 4-week clinical trial in acute schizophrenia (Leweke et al 2012) and also has been found to reduce anxiety symptoms in social phobia and following public speaking. Human data also suggest that it attenuates the cognitive impairments associated with use of the main psychoactive ingredient in cannabis. However, whether CBD may be useful in relieving symptoms and distress in patients at clinical high-risk of psychosis (CHR) has never been tested. Furthermore, how the beneficial effect of CBD on psychotic and anxiety symptoms may be mediated in the brain remains unclear.

The aim of the present study was to investigate whether short-term treatment with CBD was associated with preliminary evidence of therapeutic benefit and understand the neurocognitive mechanisms.

**Methods:**

We investigated the effects of short-term (21 days) treatment with CBD on psychotic and anxiety symptoms in 33 CHR patients, using a placebo-controlled, double-blind, parallel-arm design (CBD arm- 16; placebo arm- 17).

In the subjects who received 21 days of treatment, we used fMRI in conjunction with a verbal memory task to assess the effect of CBD relative to placebo treatment on medial temporal and striatal function.

**Results:**

Of the 33 CHR patients recruited into the trial, 31 completed treatment for 21 days. Following 21-day treatment (intention-to-treat, last observation carried forward analysis), CBD-treated (n=16) CHR patients showed a significantly greater reduction in anxiety (p=0.02) and in distress associated with psychotic symptoms (p=0.03) and a trend (p=0.14) toward greater reduction in the severity of psychotic symptoms compared to those treated with placebo (n=17) CHR patients (Figure 4). In addition, CBD was tolerated as well as placebo.

Consistent with our predictions, treatment with CBD (n=15) attenuated the engagement during verbal encoding of the parahippocampal cortex, but increased activation in the putamen in CHR patients. A similar pattern of activation was evident during verbal recall, with CBD treatment associated with increased engagement in the putamen.

**Discussion:**

Results from our proof-of-concept study suggest that 3-week treatment with CBD has beneficial effects on anxiety, attenuated psychotic symptoms and the distress associated with psychotic symptoms. They also suggest that short-term treatment with CBD modulates both medial temporal and striatal function in CHR patients, regions that are critically implicated in the CHR state. Coupled with the absence of significant adverse effects associated with CBD, a particularly important issue in relation to the treatment of CHR individuals, not all of whom develop a full-blown psychotic disorder, these data indicate that long-term treatment with CBD is likely to be efficacious in CHR patients.

